# Pim-2/mTORC1 Pathway Shapes Inflammatory Capacity in Rheumatoid Arthritis Synovial Cells Exposed to Lipid Peroxidations

**DOI:** 10.1155/2015/240210

**Published:** 2015-05-04

**Authors:** Geng Yin, Yan Li, Min Yang, Xiao-min Cen, Qi-bing Xie

**Affiliations:** ^1^Department of Rheumatology and Immunology, West China Hospital of Sichuan University, Chengdu, Sichuan 610041, China; ^2^Department of Rheumatology, The First Affiliated Hospital of Xiamen University, No. 55, Zhenhai Road, Xiamen, Fujian 361003, China

## Abstract

Rheumatoid arthritis is a systemic autoimmune disease characterized by chronic inflammation of multiple joints, with disruption of joint cartilage. The proliferation of synovial fibroblasts in response to multiple inflammation factors is central to the pathogenesis of rheumatoid arthritis. Our previous studies showed that 4-HNE may induce synovial intrinsic inflammations by activating NF-*κ*B pathways and lead to cell apoptosis. However, the molecular mechanisms of how synovial NF-*κ*B activation is modulated are not fully understood. Here, the present findings demonstrated that 4-HNE may induce synovial intrinsic inflammations by mTORC1 inactivation. While ectopic activation of mTORC1 pathway by the overexpression of Pim-2 may disrupt the initiation of inflammatory reactions and maintain synovial homeostasis, our findings will help to uncover novel signaling pathways between inflammations and oxidative stress in rheumatoid arthritis development and imply that Pim-2/mTORC1 pathway may be critical for the initiation of inflammatory reactions in human rheumatoid arthritis synovial cells.

## 1. Introduction

Rheumatoid arthritis (RA) is a chronic inflammatory disease of synovium that can lead to severe joint damage [1]. The central pathogenesis of rheumatoid arthritis is the proliferation of fibroblast-like synoviocytes (FLSs) in response to stimulators [2, 3]. During the process of FLSs proliferation, inflammatory responses are critical for rheumatoid arthritis development [4, 5]. Synovial inflammatory responses are mainly induced by products of autocrine, but also paracrine molecules produced by infiltrating mononuclear cells, such as tumor necrosis factor alpha (TNF-*α*) and interleukin (IL)-1*β*, IL-6, and IL-17 [5]. Therefore, the initiation and proceeding of inflammatory reactions might be critical for rheumatoid arthritis development.

Since inflammatory reactions are important for synovial homeostasis, the precise regulation of inflammation must be well achieved. And one of the central regulators is nuclear factor kappa beta (NF-*κ*B) [6, 7]. It has been long appreciated that NF-*κ*B is a significant transcription factor that functions in immune and inflammatory responses, stress responses, apoptosis, and differentiation. For example, NF-*κ*B plays a pivotal role in myocardial ischemia-reperfusion injury and induces many proinflammatory cytokines and chemokines which will greatly contribute to myocardial I-R injury [8]. Moreover, NF-*κ*B is also considered to act as a redox sensitive transcription factor that has been proposed to be the sensor for oxidative stress [9]. Thus, NF-*κ*B activity is important for inflammatory reactions.

Notably, among all the regulators of NF-*κ*B activation, mammalian target of rapamycin complex 1 (mTORC1) is of great significance, which is involved in differentially regulating the levels of pro- and anti-inflammatory cytokines produced by innate immune cells [10]. Studies using the mTORC1 inhibitor rapamycin have reported that mTORC1 activity plays an important role in NF-*κ*B activation and inflammation [11–13]. For example, inhibition of mTORC1 in lipopolysaccharide- (LPS-) stimulated cells has been shown to attenuate the phosphorylation of several targets of mTORC1, including p70S6K and 4E (eIF4E) binding protein 1 (4EBP1), as well as decreasing the levels of phosphorylated STAT3 (p-STAT3) [14]. In contrast, mTORC1 inhibition potently increased NF-*κ*B activity, leading to enhanced IL-12 production by LPS-stimulated cells [15]. Thus, these findings support the notion that mTORC1 pathway is a master regulator of NF-*κ*B activation and inflammation. In our previous studies, we have demonstrated that products of lipid peroxidation, 4-HNE, may induce synovial inflammations by activating NF-*κ*B pathways and lead to cell apoptosis. Pharmacological inhibition of NF-*κ*B activation may reduce the 4-HNE-mediated inflammations and subsequent cell apoptosis (unpublished data). However, how NF-*κ*B is activated under lipid peroxidation conditions is not well characterized. Considering that mTORC1 inhibition may promote NF-*κ*B activation by LPS-stimulated cells, we hypothesized that lipid peroxidation may induce NF-*κ*B activation and inflammation by inhibiting mTORC1 activity.

In the present study, we proposed to determine whether mTORC1 activity is critical for lipid peroxidation-mediated inflammation in rheumatoid arthritis synovial cells. Our findings showed that synovial mTORC1 activity was dramatically decreased by 4-HNE treatment. Moreover, we also noticed an interesting upregulation of Pim-2 kinase activity, which is a serine/threonine kinase controlling cell growth and differentiation. Overexpression of Pim-2 kinase restored the 4-HNE-mediated mTORC1 inactivation and thus led to NF-*κ*B inactivation and inflammation reduction. Our findings will help to uncover novel signaling pathways between inflammations and oxidative stress in rheumatoid arthritis development and offer new targets to rheumatoid arthritis clinical therapy.

## 2. Material and Methods

### 2.1. Chemicals and Reagents

The 4-hydroxynonenal was obtained from Biomol (Plymouth Meeting, PA, USA). Dulbecco's modified essential medium (DMEM) and fetal bovine serum (FBS) were purchased from GIBCO Invitrogen (Carlsbad, CA, USA). The following antibodies, anti-COX-2, anti-Lamin A/C, and anti-beta actin were from Santa Cruz Biotechnology (Santa Cruz, CA, USA). The Pim-2 and myc-tagged antibodies were from Millipore (Billerica, MA, USA). The pp70S6K, p70S6K, p4EBP1, 4EBP1, pBad, Bad, NF-*κ*B (p65), and GAPDH antibodies were from Cell Signaling Technology (Danvers, MA, USA). Other chemicals were of the highest purity available.

### 2.2. Cell Culture and Pharmacological Manipulations

A widely used MH7A human rheumatoid arthritis synovial cell was chosen as* in vitro* experiment system, which was obtained from Shanghai Institute of Cell Biology (Introduced from American Type Culture Collection). For Western blots and real-time PCR experiments, MH7A cells were plated in 6-well plates at 1.0 × 10^6^ cells/mL, while immunostaining at 1.0 × 10^5^ cells/mL. The cells were incubated in DMEM containing 10% FBS plus antibiotics for 24 h in 5% CO_2_ at 37°C.

For Pim-2 overexpression, the myc-tagged Pim-2 construct in MH7A cells was generated by subcloning the PCR-amplified human Pim-2 coding sequence into pRK5-myc vectors. Following transfection was carried out when the cell confluent was 80–90% using lipofectamine 2000, and cells were harvested at 24 h after transfection with lysis buffer. For 4-HNE treatment, the final concentrations of 5 *μ*M of 4-HNE were applied to these cells and then incubated for 0 to 12 h. Equivalent saline was used as internal controls. After culturing, the cells were harvested for subsequent examinations.

### 2.3. Cell Lysates Preparation and Western Blots

For Western blots, prepared cells were trypsinized and harvested, washed with PBS once, and resuspended in cell lysis buffer (PBS with 1% Triton X-100 and protease inhibitors). After brief sonication, cell lysates were centrifuged at 13,000 rpm for 5 min. Protein concentration was determined so that equivalent amounts of lysate based on protein concentration was added to an equal volume of 2x Laemmli buffer and boiled for 10 min.

As for the NF-*κ*B nuclear examinations, MH7A nuclear lysates were prepared according to the instructions of Nuclear/Cytosol Fractionation Kit (Biovision) as previously described. Briefly, MH7A cell pellets were resuspended in nuclei isolation buffer (20 mM HEPES-KOH, 100 mM KCl, 1.5 mM MgCl_2_, 1 mM EGTA, 250 mM sucrose, 1 mM phenylmethylsulfonyl fluoride, 1 mM dithiothreitol, and 10 *μ*g/mL each of aprotinin, leupeptin, and pepstatin). The cells were incubated on ice 30 min and then lysed using a homogenizer. Then, cell lysates were centrifuged (1000 ×g for 5 min), and the pellet (nuclei) and supernatant (cytosol) were collected. The nuclei were washed once in nuclei isolation buffer and pelleted by centrifugation, and the resulting supernatant was added to the cytosolic extract. For Western blot analysis, the procedure was according to the standard protocols. Finally, proteins were detected by SuperSignal enhanced chemiluminescence development (ECL) (Thermo Scientific Pierce) reagent and exposed to films (Kodak). The protein level quantification was carried out by ImageJ.

### 2.4. Immunostaining

For pp70S6K immunostaining in MH7A cells, HNE-treated cells were fixed with 4% PFA with 4% sucrose in phosphate-buffered saline (PBS) for 30 min and permeabilized with 0.25% Triton-X 100 in PBS for 5 min at room temperature. After incubated with pp70S6K primary antibody and fluorescence secondary antibody, cells were mounted onto glass slides with antifade reagent with DAPI and visualized under fluorescence microscopy.

### 2.5. Quantitative Real-Time PCR

Total RNA was extracted from tissues using TRizol reagent (Invitrogen). RNA was subjected to reverse transcription with reverse transcriptase as Manufacturer's instructions (Fermentas). Quantitative real-time PCR was performed using Bio-Rad iQ5 system, and relative gene expression was normalized to internal control as* Beta actin*. Primer sequences for SYBR Green probes of target genes are as follows:* Pim-2*: ACTCCAGGTGGCCATCAAAG and T CCATAGCAGTGCGACTTCG;* Tnf-α*: CATCTTCTCAAAATTCGAGTGACA and T GGGAGTAGACAAGGTACAACCC;* Beta actin*: GAGACCTTCAACACCCCAGC and ATGTCACGCACGATTTCCC.

### 2.6. Statistical Analysis

Data represent the mean and standard error of the mean (SEM). ANOVA tests for comparisons were performed for all statistical significance analysis using GraphPad Prism software. ^∗^ *P* < 0.05, ^∗∗^ *P* < 0.01, and ^∗∗∗^ *P* < 0.001.

## 3. Results

### 3.1. Lipid Peroxidations Inactivate mTORC1 Activity in Rheumatoid Arthritis Synovial Cells

**Figure 1:**
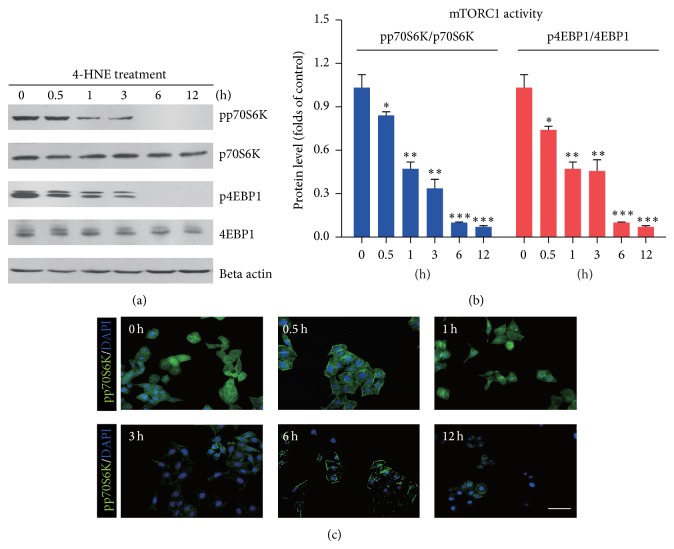
4-HNE inactivates mTORC1 activity in MH7A rheumatoid arthritis synovial cells. (a-b) Western blots and histograms showing the decreased mTORC1 activity (indicated by pp70S6K/p70S6K and p4EBP1/4EBP1) by 4-HNE treatment in MH7A synovial cells. (c) Images showing that pp70S6K signals were decreased by 4-HNE treatment in MH7A synovial cells. Green fluorescence indicates pp70S6K, and blue indicates DAPI. Bar 25 *μ*m. Results are averages of three independent experiments. Data represent mean ± SEM. ^∗^ *P* < 0.05, ^∗∗^ *P* < 0.01, and ^∗∗∗^ *P* < 0.001.

In previous studies, we have verified that products of lipid peroxidations, 4-HNE, may induce synovial intrinsic inflammations and lead to cell apoptosis (unpublished data). However, the molecular mechanisms involved in inflammatory reactions and cell apoptosis by lipid peroxidations were not fully elucidated. Considering that mTORC1 pathway is a key regulator of innate inflammatory homeostasis in several types of cells [16], we investigated mTORC1 activities by 4-HNE treatment in MH7A rheumatoid arthritis synovial cells. Biochemical results showed that, by 4-HNE treatment, the protein levels of markers of mTORC1 pathway (pp70S6K and p4EBP1) [17] were both decreased gradually as 4-HNE treatment, and the maximum folds decreased by almost 90% (6~12 h) compared to the control (Figures 1(a) and 1(b)). To confirm that reduced mTORC1 activity in MH7A cells by 4-HNE treatment, we further carried out pp70S6K immunostaining on these cells. Images showed that the pp70S6K signals (green fluorescence) also dramatically decreased by 4-HNE treatment (Figure 1(c)). Therefore, our results revealed that lipid peroxidation may inhibit mTORC1 pathway in synoviocytes, which may confer to the development of inflammations.

### 3.2. Lipid Peroxidation Activates Pim-2 Kinase Signaling in Rheumatoid Arthritis Synovial Cells

**Figure 2:**
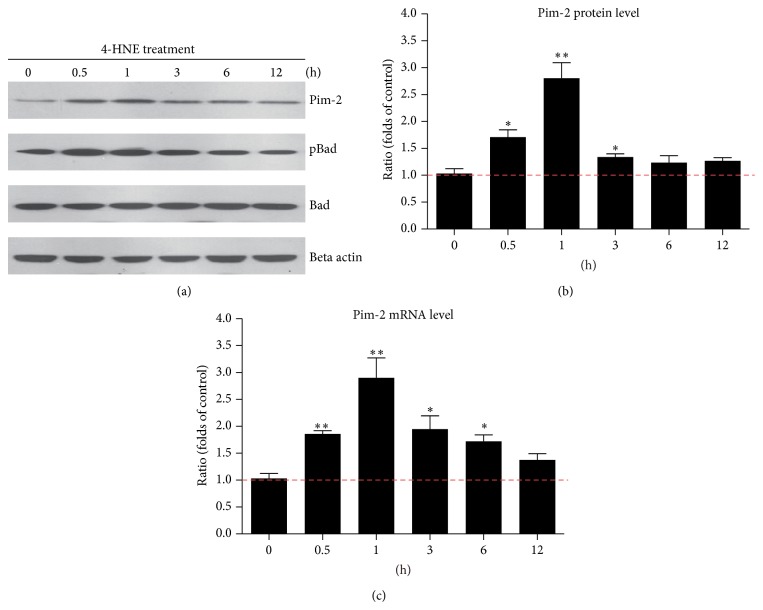
4-HNE activates Pim-2 kinase signaling in MH7A synovial cells. (a-b) Western blots and histograms showing the increased Pim-2 kinase protein levels by 4-HNE treatment in MH7A synovial cells. (c) Histograms showing that the increased Pim-2 kinase mRNA levels by 4-HNE treatment in MH7A synovial cells. Results are averages of three independent experiments. Data represent mean ± SEM. ^∗^ *P* < 0.05 and ^∗∗^ *P* < 0.01.

As for mTORC1 pathway is the master regulator cell growth, survival, and metabolism in mammalian cells [18], the decreased mTORC1 pathway by 4-HNE may induce adaptative alternations to compensate for the reduced mTORC1 activity. Pim kinase family, especially Pim-2, has been reported to be essential component of an endogenous pathway, activating mTORC1 signaling and regulating cell growth and survival [19, 20]. Thus, we examined whether Pim-2 kinase expression/activity was altered by 4-HNE treatment. Biochemical results showed that after short-term 4-HNE treatment, the protein level of endogenous Pim-2 kinase increased by 2.81-fold (1 h) compared to controls. As prolonged 4-HNE treatment, the Pim-2 protein level started to decrease, confirmed by the parallel reduction of BAD phosphorylation (a well-known Pim-2 substrate) [21] (Figures 2(a) and 2(b)). To investigate whether increased Pim-2 expressions were caused by upregulated transcriptions, we assessed the mRNA level of Pim-2. The results of quantitative real-time PCR showed that Pim-2 mRNA levels were indeed induced by 4-HNE treatment and highly correlated with the alternations of protein levels (Figure 2(c)). Thus, our findings showed that induced Pim-2 signaling may be cell intrinsic protective mechanisms against the toxicity of lipid peroxidations.

### 3.3. Pim-2 Overexpression May Partly Activate mTORC1 Pathway under 4-HNE Conditions

**Figure 3:**
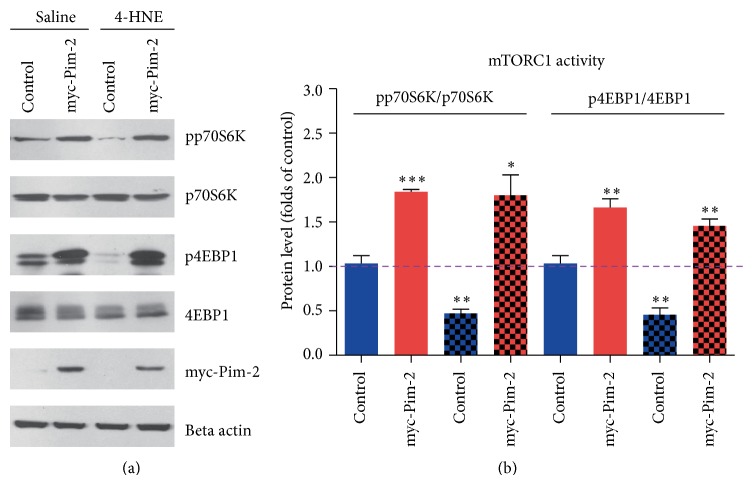
Pim-2 kinase overexpression may partly activate mTORC1 pathway under HNE conditions. (a-b) Western blots and histograms showing that Pim-2 overexpression activated mTORC1 activity (indicated by pp70S6K/p70S6K and p4EBP1/4EBP1) under basal and 4-HNE conditions. Results are averages of three independent experiments. Data represent mean ± SEM. ^∗^ *P* < 0.05, ^∗∗^ *P* < 0.01, and ^∗∗∗^ *P* < 0.001.

Since Pim-2 kinase has been reported to activate mTORC1 pathway by modulating tuberous sclerosis complex 2 (TSC2) phosphorylations [19], we proposed that upregulated Pim-2 kinase activity may partly resist 4-HNE-mediated mTORC1 inactivation. To examine how Pim-2 participates in mTORC1 activation under oxidative stress, we constructed an myc-tagged Pim-2 vector to the overexpression of Pim-2 in MH7A synovial cells and investigated the mTORC1 signaling alternations. Biochemical results showed that although 4-HNE treatment may decrease p70S6K and 4EBP1 phosphorylations, Pim-2 overexpression may constitutively maintain high phosphorylations of p70S6K and 4EBP1 under both basal and 4-HNE conditions (Figures 3(a) and 3(b)). These results clearly indicate that the overexpression of Pim-2 may promote constitutive mTORC1 activation under oxidative stress, which may contribute to maintenance of cell homeostasis.

### 3.4. Pim-2 Mediated mTORC1 Activation Inhibits 4-HNE-Induced Inflammation in Rheumatoid Arthritis Synovial Cells

**Figure 4:**
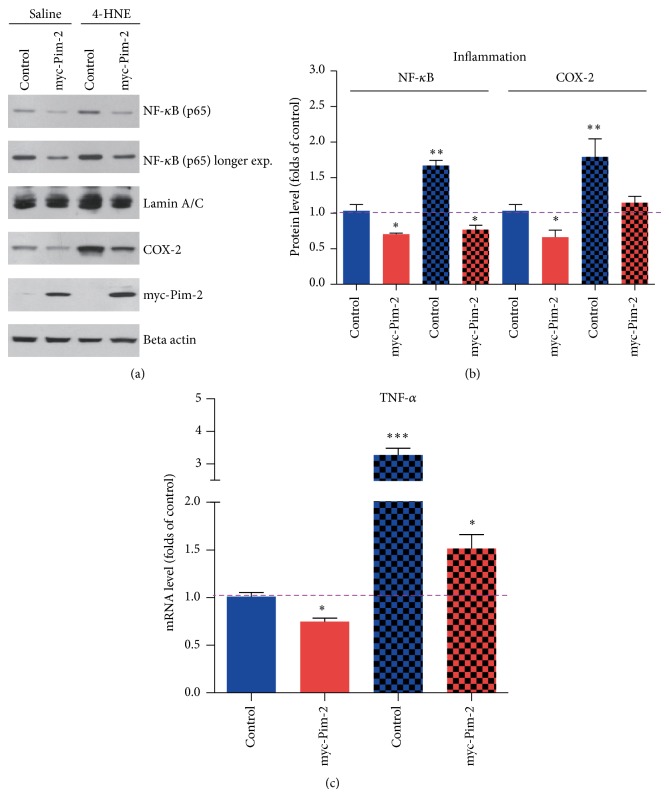
Pim-2 mediated mTORC1 activation inhibits HNE-induced inflammation in MH7A synovial cells. (a-b) Western blots and histograms showing that Pim-2 overexpression inhibited NF-*κ*B nuclear localizations and COX-2 expressions under basal and 4-HNE conditions. (c) Histograms showing that Pim-2 overexpression inhibited TNF-*α* transcriptions under basal and 4-HNE conditions. Results are averages of three independent experiments. Data represent mean ± SEM. ^∗^ *P* < 0.05, ^∗∗^ *P* < 0.01, and ^∗∗∗^ *P* < 0.001.

Oxidative stress induced inflammation is critical for rheumatoid arthritis development [22, 23]. In our previous studies, we have shown that 4-HNE could induce synovial intrinsic inflammations by activating NF-*κ*B and COX-2 pathways (unpublished data). Moreover, mTORC1 activity has been proved to be critical for the inflammatory balance. Therefore, we assumed that Pim-2 might mediate mTORC1 activation and may inhibit inflammation reactions caused by lipid peroxidation. Thus, we examined the inflammation markers of NF-*κ*B and COX-2 in Pim-2 overexpressed MH7A cells. The results showed that the overexpression of Pim-2 may inhibit NF-*κ*B nuclear localization of the p65 subunit under both basal and 4-HNE conditions, which indicated that the overexpression of Pim-2 may inhibit NF-*κ*B signaling. Moreover, the induced COX-2 expression by 4-HNE might be also disrupted by the overexpression of Pim-2, which confirmed that the overexpression of Pim-2 may inhibit the initiation of inflammatory reactions (Figures 4(a) and 4(b)). To further confirm that the overexpression of Pim-2 may contribute to the inflammatory blocking in MH7A cells, we examined the mRNA level of canonical inflammation factor TNF-*α*. The results also showed that the overexpression of Pim-2 may inhibit 4-HNE induced TNF-*α* transcriptions (Figure 4(c)). All these findings showed that Pim-2/mTORC1 pathway may play a vital role in protection against lipid peroxidation induced inflammations.

## 4. Discussion

**Figure 5:**
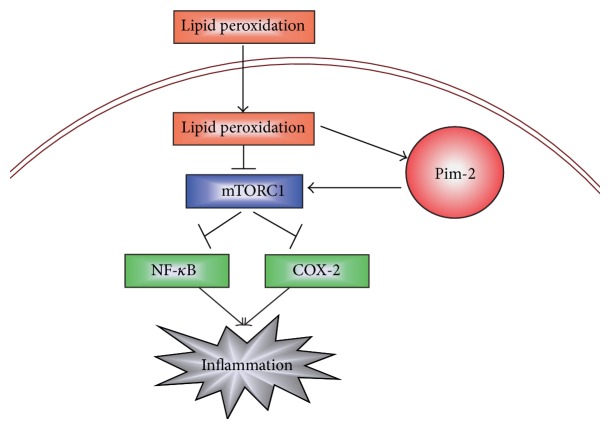
A model showing that Pim-2/mTORC1 pathway may be critical for the initiation of inflammatory reactions mediated by lipid peroxidation in human rheumatoid arthritis synovial cells.

In the present study, we reveal a novel mechanism to clarify how inflammation is precisely regulated in human rheumatoid arthritis synovial cells. The present findings demonstrated that products of lipid peroxidation, 4-HNE, may induce synovial intrinsic inflammations by activating NF-*κ*B pathways which may be mediated by mTORC1 inactivation. However, ectopic activation of mTORC1 pathway by the ove-expression of Pim-2 may disrupt the initiation of inflammatory reactions and maintain synovial homeostasis (Figure 5). Our work will help to uncover novel signaling pathways between inflammations and oxidative stress in rheumatoid arthritis development and offer new targets to rheumatoid arthritis clinical therapy.

Rheumatoid arthritis (RA) is a chronic, systemic, and inflammatory autoimmune disease, targeting the synovial tissues [24]. Fibroblast-like synoviocytes (FLSs) play a central role in the formation of rheumatoid arthritis pannus [25]. Recent advances in understanding the networks that are responsible for the synovial inflammations in rheumatoid arthritis have led to the successful use of therapies [26, 27]. Yet, the regulatory mechanisms of how inflammation is precisely controlled remain incompletely understood. Here, we demonstrate that synovial Pim-2/mTORC1 pathway couples the oxidative stress and inflammations. Under oxidative stress conditions (lipid peroxidation), mTORC1 activity was downregulated, which may be responsible for the initiation of inflammatory reactions, such as NF-*κ*B nuclear translocations, COX-2 expressions, and inflammation factors releases. On the other hand, to precisely modulate inflammatory reactions, oxidative stress may activate Pim-2 kinase signaling to maintain appropriate mTORC1 activity. Therefore, once Pim-2 activity is ectopic upregulated (e.g., Pim-2 overexpression, etc.), mTORC1 activity may be restored under 4-HNE conditions, which disrupts the synovial inflammations. Thus, Pim-2/mTORC1 pathway may be critical for the coupling of oxidative stress and synovial inflammation.

In contrast to many other kinases whose activities are tuned by phosphorylation status, the Pim-2 kinase is constitutively active and lacks regulatory domains. Instead, Pim-2 kinase is tightly regulated at both transcriptional and translational levels [19]. The signals that induce Pim-2 gene expression are diverse, including various cytokines, growth factors, and mitogenic stimuli in different cell types. Moreover, Pim-2 could modulate mTORC1 activity by directly phosphorylating TSC2 on Ser-1798 and relieves the suppression of TSC2 on mTORC1 [19]. Here, we further showed that Pim-2 expression may be induced products of lipid peroxidation, to compensate for the mTORC1 activity under oxidative stress conditions. Thus, further in-depth studies on how Pim-2 senses oxidative stress and is transcriptionally upregulated in human rheumatoid arthritis synovial cells might not only help to understand inflammatory reactions and/or synovial homeostasis, but possibly also uncover novel signaling pathways between inflammations and oxidative stress.

## 5. Conclusion

The present findings suggested that lipid peroxidation-mediated mTORC1 inactivation may be essential for the synovial inflammation. While ectopic activation of Pim-2 signaling may partly restore mTORC1 activity under lipid peroxidation conditions, leading to inflammation blocking, our findings imply that Pim-2/mTORC1 pathway may be critical for the initiation of inflammatory reactions and cell homeostasis in human rheumatoid arthritis synovial cells.
